# The Decline in Hydrocodone/Acetaminophen Prescriptions in Emergency Departments in the Veterans Health Administration Between 2009 to 2015

**DOI:** 10.5811/westjem.2016.5.29924

**Published:** 2016-06-15

**Authors:** Michael A. Grasso, Zachary D.W. Dezman, Angela C. Comer, David A. Jerrard

**Affiliations:** * University of Maryland, Department of Emergency Medicine, Baltimore, Maryland; † University of Maryland, National Study Center for Trauma and EMS, Maryland

## Abstract

**Introduction:**

The purpose of the study was to measure national prescribing patterns for hydrocodone/acetaminophen among veterans seeking emergency medical care, and to see if patterns have changed since this medication became a Schedule II controlled substance.

**Methods:**

We conducted a retrospective cohort study of emergency department (ED) visits within the Veterans Health Administration (VA) between January 2009 and June 2015. We looked at demographics, comorbidities, utilization measures, diagnoses, and prescriptions.

**Results:**

During the study period, 1,709,545 individuals participated in 6,270,742 ED visits and received 471,221 prescriptions for hydrocodone/acetaminophen (7.5% of all visits). The most common diagnosis associated with a prescription was back pain. Prescriptions peaked at 80,776 in 2011 (8.7% of visits), and declined to 35,031 (5.6%) during the first half of 2015 (r=−0.99, p<0.001). The percentage of hydrocodone/acetaminophen prescriptions limited to 12 pills increased from 22% (13,949) in 2009 to 31% (11,026) in the first half of 2015. A prescription was more likely written for patients with a pain score≥7 (OR 3.199, CI [3.192–3.205]), a musculoskeletal (OR 1.622, CI [1.615–1.630]) or soft tissue (OR 1.656, CI [1.649–1.664]) diagnosis, and those below the first quartile for total ED visits (OR 1.282, CI [1.271–1.293]) and total outpatient ICD 9 codes (OR 1.843, CI [1.833–1.853]).

**Conclusion:**

Hydrocodone/acetaminophen is the most frequently prescribed ED medication in the VA. The rate of prescribing has decreased since 2011, with the rate of decline remaining unchanged after it was classified as a Schedule II controlled substance. The proportion of prescriptions falling within designated guidelines has increased but is not at goal.

## INTRODUCTION

### Background

In the late 1990s, there was a growing belief that physicians were under-treating pain.^1^,^2^,^3^ Some investigators demonstrated that the risk of addiction was less than what had been perceived,^4^ so national professional medical societies and the Joint Commission on Accreditation of Healthcare Organizations (JCAHO) began advocating aggressive pain control.^5^,^6^,^7^ The Veterans Health Administration (VA) followed suit, launching the “Pain as the Fifth Vital Sign” campaign in 1998.^8^,^9^ Since that time, the United States has seen a three-fold increase in the use of pain medications 10 and is the world’s biggest consumer of prescription opioid pain medications. Roughly 260 million opioid prescriptions were written in 2012.^11^ It is estimated that 2.1 million people in the U.S. are currently abusing prescription opioid pain medications 12 and that 23,000 unintentional deaths were attributable to them in 2013.^13^

Hydrocodone is a semisynthetic opioid medication, originally derived from codeine. It was developed in the 1920s and approved for use by the Food and Drug Administration in 1943. The combination of hydrocodone/acetaminophen was approved in 1983. Hydrocodone-containing products are now the most frequently prescribed opioids in the U.S., with the combination of hydrocodone/acetaminophen being the most popular.^14^ The U.S. accounts for 99% of all hydrocodone prescriptions worldwide.^15^

### Importance

In 2012, the Centers for Disease Control and Prevention linked the rise in prescription opioid use to an increase in drug overdoses and opioid abuse.^16^–^19^ In response, many agencies and authors now advocate changes in opioid-prescribing habits,^12^,^20^–^22^ especially in the ED.^23^,^24^ This change imposes important challenges, since pain is the most common presenting complaint in the ED,^25^–^28^ and a significant number of ED visits result in an opioid pain prescription.^29^,^30^

Many jurisdictions now advocate that emergency physicians limit opioid prescriptions to a three-day supply of a short-acting medication.^31^,^32^ A recent cross-sectional study reported that ED patients received an average of 17 pills of short-acting pain medications per prescription.^31^ Concerns about the rising use of hydrocodone specifically led the U.S. Drug Enforcement Agency to reclassify it as a Schedule II drug, effective October 6th, 2014.^33^ The VA launched its own safety campaign on October 1st, 2014 (VA Opioid Safety Initiative), to teach providers and patients about appropriate opioid use.^34^

### Goals of This Investigation

This observational study sought to measure national trends in prescribing patterns for hydrocodone/acetaminophen in VA EDs, and to determine if prescribers are following appropriate prescription guidelines. A secondary objective was to see if prescribing patterns have changed since this medication became a Schedule II controlled substance.

## METHODS

### Study Design and Setting

The VA is an integrated healthcare system encompassing more than 150 hospitals and 800 community-based outpatient clinics. In 2013, the system provided comprehensive care to 8.9 million veterans through 86 million outpatient visits and 700,000 hospital admissions.^35^ A single electronic health record system is used across the VA system by more than 50,000 providers. It captures demographics, diagnostic codes, outpatient visits, hospital admissions, patient orders, vital signs, laboratory test results, inpatient and outpatient pharmacy data, clinical consults, immunizations, mental health screening, associated physicians, payment information, progress notes, radiology reports, procedure reports, images, and clinical narratives.

In this retrospective cohort study, we analyzed data across the entire VA population. Data for our study were obtained from the VA Informatics and Computing Infrastructure (VINCI), which maintains the national VA clinical repository and makes these data available to researchers within the VA system.^36^ This study was supported with resources and facilities at the Baltimore VA Medical Center and the Veterans Affairs Informatics and Computing Infrastructure. Regulatory approval was obtained through the University of Maryland School of Medicine and the Baltimore VA Medical Center. Our research protocol included an informed-consent waiver.

### Selection of Participants

We developed a study cohort using data from the national VA repository. Eligible participants included all veterans who received emergency medical care within the VA system in the 78-month period between January 1, 2009, and June 30, 2015. Patients were excluded from the study if their age, gender, location, or diagnosis was missing from their medical record (292,390 patients) or if they were born after 1996 (624 patients).

### Methods and Measurements

For each participant, we collected information on demographics, comorbidities, the ED encounter, and utilization of medical services. Demographic measures were age, gender, and ethnicity.

Encounter measures were visit date, initial pain score, and diagnosis. The diagnoses for each encounter were organized into six categories by ICD-9 code as musculoskeletal, soft tissue, trauma, oncologic, psychiatric, or medical. The quantity and days of hydrocodone/acetaminophen prescribed were collected, and noted as to whether they followed the appropriate prescribing guidelines of no more than twelve pills and a three-day supply.

Utilization measures were the total number of ICD-9 codes from outpatient clinic visits, total number of ED visits, and total opioid medication doses prescribed to each patient between January 1, 2009, and July 15, 2015. The number of ED visits is commonly used, and provides an unbiased measure of how often a patient interacts with the medical system.^37^ The number of outpatient ICD-9 codes provides a weighted measure of visits, as we would expect patients with multiple comorbidities to require frequent follow-up visits. Total doses of opioids provides a similar, unbiased estimate of the amount of pain medications a patient receives.

Comorbidity measures included 12 medical and mental health diagnoses, a dual diagnosis of a mental health issue and a substance abuse issue and Comorbidity-Polypharmacy Score (the sum of the pre-visit medications with the number of comorbid conditions).^38^ The medical and mental health diagnoses were cardiovascular disease, type 2 diabetes, chronic kidney disease, chronic obstructive pulmonary disease, osteoarthritis, chronic pain, depression, post-traumatic stress disorder, bipolar disorder, schizophrenia, substance abuse, and alcohol abuse. The chronic pain conditions were central pain syndrome, chronic fatigue syndrome, chronic headache, chronic interstitial cystitis, chronic pain, fibromyalgia, irritable bowel syndrome, psychogenic pain, and temporomandibular joint disorder.

### Analysis

We summarized the characteristics of the participants by age, gender, ethnicity, comorbidity, utilization factors, date, and diagnosis. In addition, for each ED visit, characteristics were organized by year to identify trends in prescribing habits. We used the chi-square test, with 95% confidence intervals, to examine differences among characteristics between groups. Pearson’s correlation coefficient was used to evaluate prescribing trends by year. Multivariable logistic regression was used to determine the characteristics that best predicted who received a prescription for hydrocodone/acetaminophen.

## RESULTS

### Study Population

The ED cohort included 1,709,545 individuals who met the inclusion criteria. They accounted for 6,270,742 ED visits between January 1, 2009, and June 30, 2015. Their median age was 58 years (interquartile range, [48–66]), 91% were male, and 59% were Caucasian. Across all ED visits, the average CPS was 28 (median 24, interquartile range, [14–37]), with 22% having coronary artery disease, 32% having diabetes, and 13% having at least one chronic pain diagnosis. Roughly 36% had depression, 20% had post-traumatic stress disorder, and 21% had a dual diagnosis of a mental health condition and a substance abuse problem (Table 1). All demographic values had significant p values (less than 0.001) when comparing all ED visits to those visits where hydrocodone/acetaminophen was prescribed.

The most frequently prescribed medication for all ED visits was hydrocodone/acetaminophen (471,221 [7.5% of ED visits]), followed by ibuprofen (247,460 [4.0%]) and prednisone (245,990 [3.9%]) (Table 2). The most common ED diagnoses were back pain (264,589 [4.2%]), chest pain (233,437 [3.7%]), and skin infections (187,765 [3.0%]). The most common diagnoses among those receiving a prescription for hydrocodone/acetaminophen were back pain (76,131 [16.2%]), arthropathy (63,550 [13.5%]), and oral/dental issues (25,577 [5.4%]) (Table 3).

### Prescribing Trends

**Figure 1:**
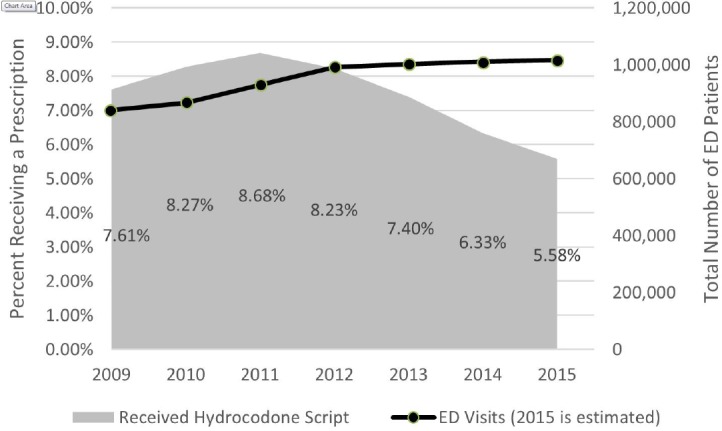
Hydrocodone/acetaminophen prescribing percentages.

Within the VA system, the annual number of prescriptions for hydrocodone/acetaminophen peaked at 80,776 in 2011, associated with 8.7% of ED visits during that year. The number has shown a downward trend since that time, decreasing to 63,991 (6.33% of visits) in 2014 and 35,031 (5.6% of visits) for the first half of 2015. Beginning at the peak in 2011, the data show a strong linear correlation with time (r =−0.99 [p <0.001]). The number of ED visits increased from 841,256 in 2009 to 1,010,773 in 2014 and is estimated to be 1,015,968 in 2015 (Figure 1).

**Figure 2:**
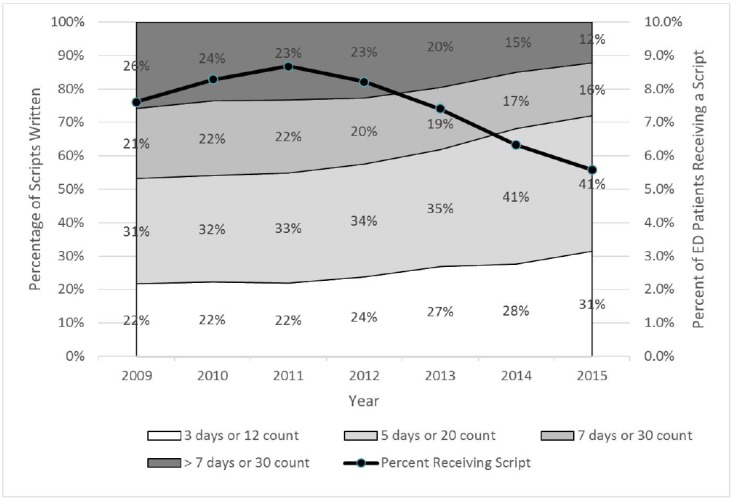
Hydrocodone/acetaminophen prescribing practices. *ED,* emergency department

Overall, the average number of pills prescribed was 32 (standard deviation 38), with a median of 20 (interquartile range, [15–30]). The percentage of prescriptions limited to twelve and a three-day supply increased from 22% (13,949) in 2009 to 31% (11,026) for the first half of 2015. A similar increase was noted for prescriptions limited to 13 to 20 pills: from 31% (20,166) in 2009 to 41% (5,847) during the first half of 2015. For the same years, prescriptions of 21 to 30 pills decreased from 21% (13,412) to 16% (2,168), and prescriptions over 30 pills decreased from 26% (16,526) to 12% (4,266) (Figure 2).

### Predictors of Hydrocodone/Acetaminophen Prescribing

The predictive factors found by our logistic regression model are shown in Table 4. The factors associated with patients less likely to get a prescription for hydrocodone/acetaminophen were being 66 years or older (the third quartile for age, OR 0.679, CI [0.670–0.688]), visiting the ED 15 or more times during their tenure within the VA system (third quartile for ED visits, OR 0.831, CI [0.822–0.840]), and having more than 766 total outpatient clinic ICD-9 codes (third quartile, OR 0.663, CI [0.653–0.672]). Other patients who were less likely to get a prescription were those with a CPS below 14 (first quartile, OR 0.836, CI [0.826–0.847]); those with a dual mental health/substance abuse diagnosis (OR 0.972, CI [0.959–0.985]); or those with an ED diagnosis related to trauma (OR 0.813, CI [0.799–0.827]), cancer (OR 0.7–0.46, CI [0.738–0.753]), or a psychiatric issue (OR 0.950, CI [0.943–0.957]).

A prescription for hydrocodone/acetaminophen was more likely to be written for males (OR 1.112, CI [1.100–1.123]); Caucasians (OR 1.213, CI [1.201–1.220]); those with an initial pain score of 7 or higher (OR 3.199, OR [3.192–3.205]); and those with a musculoskeletal (OR 1.622, CI [1.615–1.630]), soft tissue (OR 1.656, CI [1.649–1.664]), or medical (OR 1.361, CI [1.354–1.367]) diagnosis in the ED. Those with a CPS between the first and third quartiles were also more likely to get a prescription (OR 1.062, CI [1.056–1.068]). In addition, a prescription was more likely for those who received at least 1,012 total opioid medication doses during their tenure within the VA system (third quartile, OR 1.506, CI [1.499–1.513]), for those who visited the ED three times or less during their tenure within the VA system (first quartile, OR 1.282, CI [1.271–1.293]), or for those having 160 or fewer total outpatient clinic ICD-9 codes (first quartile, OR 1.843, CI [1.833–1.853]).

## DISCUSSION

In this retrospective study of more than six million ED visits within the VA system, hydrocodone/acetaminophen was the most frequently prescribed medication. Because of its heavy and widespread utilization in the U.S., this medication has come under scrutiny. In our veteran population, for those seeking emergency medical care, prescriptions for hydrocodone/acetaminophen were written almost twice as often as for the next most common drug, ibuprofen. In fact, five of the 10 most prescribed medications given through the ED target pain relief. Back pain and arthropathy were the most common diagnoses for ED visits during which a prescription for hydrocodone/acetaminophen was given.

In the time of interest in this study, the percentage of VA patients receiving hydrocodone/acetaminophen scripts in the ED peaked in 2011. This coincides with the determination by the CDC that prescription opioids were the primary cause of a spike in drug-related deaths and predates the reclassification of hydrocodone/acetaminophen as a Schedule II controlled substance by the Drug Enforcement Agency, and predates the VA’s Opioid Safety Initiative. Overall, both the number of tablets given at each ED visit and the proportion of patients receiving a prescription have decreased, despite the increasing number of ED visits. The rate of decrease has remained constant since 2011 and did not change after the reclassification in October 2014. The proportion of prescriptions consistent with the latest best-practice recommendation of no more than twelve pills and a three-day supply increased (from 22% to 29%), while the proportion of scripts for large numbers of pills decreased by more than half (from 26% to 12%) over the same period. These important improvements are likely due to the efforts of many professional societies, but the numbers are still not at goal.

The demographics of our study group match those of other studies of the Veterans Administration population. Table 1 shows a slight increase in the proportion of Caucasian females receiving prescriptions. Our logistic regression model revealed that patients who were male (OR 1.112) and Caucasian (OR 1.213) were more likely to get scripts for hydrocodone/acetaminophen than females or other ethnicities. This finding warrants further investigation that might suggest a potential bias among the many confounding factors. It is notable that, due to our large study size, we had sufficient power to detect small effects that may not be clinically significant.

Patients who presented in severe pain (with a pain score of 7 or higher) and those who had a musculoskeletal, soft tissue, or medical diagnosis were more likely to receive a hydrocodone/acetaminophen prescription. Patients with cancer or a trauma diagnosis or with a history of chronic pain were less likely to receive a prescription for this drug. This observation might be the result of a substitution effect, that is, patients with cancer or traumatic injuries received different pain medications. With the exception of schizophrenia, psychiatric comorbidities had very little impact on prescribing habits. In addition, patients with an alcohol or a substance abuse diagnosis received prescriptions at very nearly the same rate. There also appears to be a population receiving this medication that is young, visits EDs and outpatient clinics infrequently, and is relatively healthy (Table 4).

## LIMITATIONS

This study is retrospective and therefore subject to selection bias, misclassification, and confounding. Our results can only show associations, and not causation. We defined our population using administrative data and ICD-9 codes. We analyzed the veteran population, a nationwide cohort of more than six million ED visits from more than 150 hospitals across the U.S.

We did not include prescriptions filled at pharmacies outside the VA. However, the VA is designed to be a contained system, with integrated pharmacy, inpatient, and outpatient services. This analysis focused on hydrocodone-acetaminophen, and did not investigate potential substitution with other less-commonly prescribed opioid medications.

## CONCLUSION

In summary, hydrocodone/acetaminophen was the most frequently prescribed ED medication within the VA system in this national sample of more than six million ED visits between 2009 and 2015. The rate of prescribing this drug has decreased since 2011. The proportion of prescriptions falling within guideline-designated safe prescribing habits increased over the same period. Relatively healthy patients, who less frequently visit the ED or outpatient clinics, with severe pain but without chronic pain, and with musculoskeletal or soft tissue diagnoses, tend to receive scripts more often than others.

## Figures and Tables

**Table 1:** Demographic and clinical characteristics of the ED cohort.

Characteristic	All ED visits	HD/A prescribed
Demographics		
Study sample, n (%)	6,270,742	471,221 (7.5%)
Male, n (%)	5,715,263 (91.1%)	426,785 (90.6%)
Age, median years (IQR)	58 (48–66)	54 (44–62)
Ethnicity, n (%)		
Caucasian	3,712,661 (59.2%)	293,393 (62.3%)
African	1,730,065 (27.6%)	125,530 (26.6%)
Hispanic	370,401 (5.9%)	18,898 (4.0%)
Asian	30,401 (0.5%)	2,287 (0.5%)
Other/unknown	426,915 (6.8%)	31,113 (6.6%)
Comorbid conditions, n (%)		
Cardiovascular disease	1,364,953 (21.8%)	83,081 (17.6%)
Type 2 diabetes	1,977,908 (31.5%)	132,843 (28.2%)
Chronic kidney disease	748,134 (11.9%)	42,786 (9.1%)
Chronic obstructive pulmonary disease	1,141,558 (18.2%)	70,427 (14.9%)
Osteoarthritis	1,752,200 (27.9%)	135,904 (28.8%)
Chronic pain	828,567 (13.2%)	74,694 (15.9%)
Depression	2,266,675 (36.1%)	183,372 (38.9%)
Post-traumatic stress disorder	1,277,666 (20.4%)	109,635 (23.3%)
Bipolar	1,323,827 (21.1%)	104,486 (22.2%)
Schizophrenia	317,835 (5.1%)	14,464 (3.1%)
Substance abuse	1,501,786 (23.9%)	120,275 (25.5%)
Alcohol abuse	1,365,015 (21.8%)	101,986 (21.6%)
Comorbidity indexes		
Comorbidity-polypharmacy index, median (IQR)	24 (14–37)	23 (14–35)
Charlson index, median (IQR)	1 (0–2)	1 (0–3)
Dual diagnosis, n (%)	1,325,975 (21.1%)	104,221 (22.1%)
Utilization		
Total ED visits, median (IQR)	7 (3–14)	7 (3–15)
Total outpatient clinic ICD 9 codes, median (IQR)	313 (129–632)	389 (160–766)
Total narcotic doses, median (IQR)	392 (90–2106)	170 (20–1012)
Initial pain score, median (IQR)	8 (5–9)	4 (0–7)
Diagnosis, n (%)		
Musculoskeletal	3,520,147 (56.1%)	332,034 (70.5%)
Soft tissue	2,322,551 (37.0%)	170,826 (36.3%)
Trauma	3,055,676 (48.7%)	267,433 (56.8%)
Cancer	1,051,614 (16.8%)	24,968 (5.3%)
Psychiatric	2,056,036 (32.8%)	133,399 (28.3%)
Medical	5,681,522 (90.6%)	407,142 (86.4%)

*ED,* emergency department; *HD/A,* hydrocodone/acetaminophen

**Table 2:** Most frequently prescribed medications in the ED cohort.

Medication	Count
Hydrocodone/acetaminophen	471,224
Ibuprofen	247,460
Prednisone	245,990
Albuterol	230,602
Azithromycin	194,010
Cyclobenzaprine	162,929
Tramadol	158,027
Naproxen	154,006
Omeprazole	151,325
Amoxicillin/clavulanate	140,370

*ED,* emergency department

**Table 3:** Top diagnoses resulting in a hydrocodone/acetaminophen prescription.

Diagnosis	ICD-9 codes	ED visits	No. of prescriptions
Back pain	724	264,589 (4.2%)	76,131 (16.2%)
Arthropathy	710–719	281,336 (4.5%)	63,550 (13.5%)
Oral, dental	520–529	27,730 (0.4%)	25,577 (5.4%)
Skin infection	680–686	187,765 (3.0%)	23,291 (4.9%)
Abdominal pain	789	156,745 (2.5%)	16,616 (3.5%)
Chest pain	786.5	233,437 (3.7%)	13,593 (2.9%)
Neck pain	723	47,461 (0.8%)	12,038 (2.6%)
Nephrolithiasis	592	32,728 (0.5%)	11,795 (2.5%)
Gout	274	42,351 (0.7%)	9,771 (2.1%)
Headache	784, 339	85,502 (1.4%)	7,732 (1.6%)

*ED,* emergency department

**Table 4:** Hydrocodone/acetaminophen prescribing predictors.

Characteristic	Odds ratio	Lower CI	Upper CI	P-value
Demographics				
Age, ≤48 years (Q1)	1.248	1.240	1.256	<0.001
Age, >48 and <66 years (Q1–Q3)	1.079	1.073	1.085	<0.001
Age, ≥66 years (Q3)	0.679	0.670	0.688	<0.001
Male	1.112	1.100	1.123	<0.001
Caucasian	1.213	1.207	1.220	<0.001
Comorbidities				
Cardiovascular disease	0.946	0.937	0.954	<0.001
Type 2 diabetes	0.988	0.981	0.996	0.002
Chronic kidney disease	0.929	0.918	0.940	<0.001
Chronic obstructive pulmonary disease	0.909	0.900	0.918	<0.001
Osteoarthritis	0.982	0.975	0.989	<0.001
Chronic pain	0.952	0.943	0.960	<0.001
Depression	1.026	1.019	1.034	<0.001
Post-traumatic stress disorder	1.101	1.093	1.109	<0.001
Bipolar	0.963	0.955	0.972	<0.001
Schizophrenia	0.757	0.739	0.774	<0.001
Substance abuse	1.050	1.039	1.061	<0.001
Alcohol abuse	0.991	0.980	1.001	0.075
Comorbidity Indexes				
Comorbidity-polypharmacy index, ≤14 (Q1)	0.836	0.826	0.847	<0.001
Comorbidity-polypharmacy index, >14 and <37 (Q1–Q3)	1.062	1.056	1.068	<0.001
Comorbidity-polypharmacy index, ≥37 (Q3)	1.010	1.001	1.020	0.035
Dual diagnosis	0.972	0.959	0.985	<0.001
Utilization				
Total ED visits, ≤3 (Q1)	1.282	1.271	1.293	<0.001
Total ED visits, >3 and <15 (Q1–3)	1.039	1.033	1.046	<0.001
Total ED visits, ≥15 (Q3)	0.831	0.822	0.840	<0.001
Total outpatient clinic ICD 9 codes, ≤160 (Q1)	1.843	1.833	1.853	<0.001
Total outpatient clinic ICD 9 codes, >160 and <389 (Q1–Q3)	0.987	0.981	0.994	<0.001
Total outpatient clinic ICD 9 codes, ≥766 (Q3)	0.663	0.653	0.672	<0.001
Total narcotic doses, ≤20 (Q1)	0.132	0.116	0.147	<0.001
Total narcotic doses, >20 and <1012 (Q1–Q3)	1.366	1.360	1.372	<0.001
Total narcotic doses, ≥1012 (Q3)	1.506	1.499	1.513	<0.001
Initial pain score of 7 or higher	3.199	3.192	3.205	<0.001
Diagnosis				
Musculoskeletal	1.622	1.615	1.630	<0.001
Soft tissue	1.656	1.649	1.664	<0.001
Trauma	0.813	0.799	0.827	<0.001
Cancer	0.746	0.738	0.753	<0.001
Psychiatric	0.950	0.943	0.957	<0.001
Medical	1.361	1.354	1.367	<0.001

*ED,* emergency department
